# Role of AE2 for pH_i_ regulation in biliary epithelial cells

**DOI:** 10.3389/fphys.2013.00413

**Published:** 2014-01-17

**Authors:** Axel R. Concepcion, María Lopez, Alberto Ardura-Fabregat, Juan F. Medina

**Affiliations:** Division of Gene Therapy and Hepatology, Center for Applied Medical Research (CIMA), School of Medicine, University of Navarra, and CiberehdPamplona, Spain

**Keywords:** bile flow, biliary HCO^−^_3_ secretion, cholangiocytes, Cl^−^/HCO^−^_3_ anion exchange, primary biliary cirrhosis

## Abstract

The Cl^−^/HCO^−^_3_anion exchanger 2 (AE2) is known to be involved in intracellular pH (pH_i_) regulation and transepithelial acid-base transport. Early studies showed that *AE2* gene expression is reduced in liver biopsies and blood mononuclear cells from patients with primary biliary cirrhosis (PBC), a disease characterized by chronic non-suppurative cholangitis associated with antimitochondrial antibodies (AMA) and other autoimmune phenomena. Microfluorimetric analysis of the Cl^−^/HCO^−^_3_ anion exchange (AE) in isolated cholangiocytes showed that the cAMP-stimulated AE activity is diminished in PBC compared to both healthy and diseased controls. More recently, it was found that miR-506 is upregulated in cholangiocytes of PBC patients and that AE2 may be a target of miR-506. Additional evidence for a pathogenic role of AE2 dysregulation in PBC was obtained with *Ae2*^−/−^_*a,b*_ mice, which develop biochemical, histological, and immunologic alterations that resemble PBC (including development of serum AMA). Analysis of HCO^−^_3_ transport systems and pH_i_ regulation in cholangiocytes from normal and *Ae2*^−/−^_*a,b*_ mice confirmed that AE2 is the transporter responsible for the Cl^−^/HCO^−^_3_exchange in these cells. On the other hand, both *Ae2*^+/+^_*a,b*_ and *Ae2*^−/−^_*a,b*_ mouse cholangiocytes exhibited a Cl^−^-independent bicarbonate transport system, essentially a Na^+^-bicarbonate cotransport (NBC) system, which could contribute to pH_i_ regulation in the absence of AE2.

## Introduction

Intracellular pH (pH_i_) regulation plays a critical role for most cellular processes and functions. Activation by environmental stimuli, DNA synthesis and cell proliferation, apoptosis, oxidative stress and cell metabolism are accompanied by changes in pH_i_ (Gerson et al., 1982; Moolenaar et al., 1983; Burns and Rozengurt, 1984; Lagadic-Gossmann et al., 2004; Mulkey et al., 2004; Cardone et al., 2005). To minimize cytosolic pH disturbances, cells employ not only their intrinsic buffering capacity but have also a variety of ion carriers at the plasma membrane that maintain the pH_i_ within a narrow physiological range (Boron et al., 2009; Casey et al., 2010). These include channels, pumps, exchangers, and cotransporters, all of which orchestrate the input and output of acid/base ions H^+^ and HCO^−^_3_. In this review, the major membrane ion carriers that contribute to the regulation of pH_i_ in cholangiocytes (the biliary epithelial cells lining intrahepatic bile ducts) are described. A particular attention is paid to the anion exchanger 2 (AE2, also Slc4A2), a pH regulatory protein that is highly activated upon increased pH_i_ (Stewart et al., 2001). AE2 is efficiently used by cholangiocytes to execute biliary HCO^−^_3_ secretion and its dysfunction is seemingly involved in the pathogenesis of primary biliary cirrhosis (PBC).

## Membrane ion carriers that regulate pH_i_ in cholangiocytes

**Figure 1:**
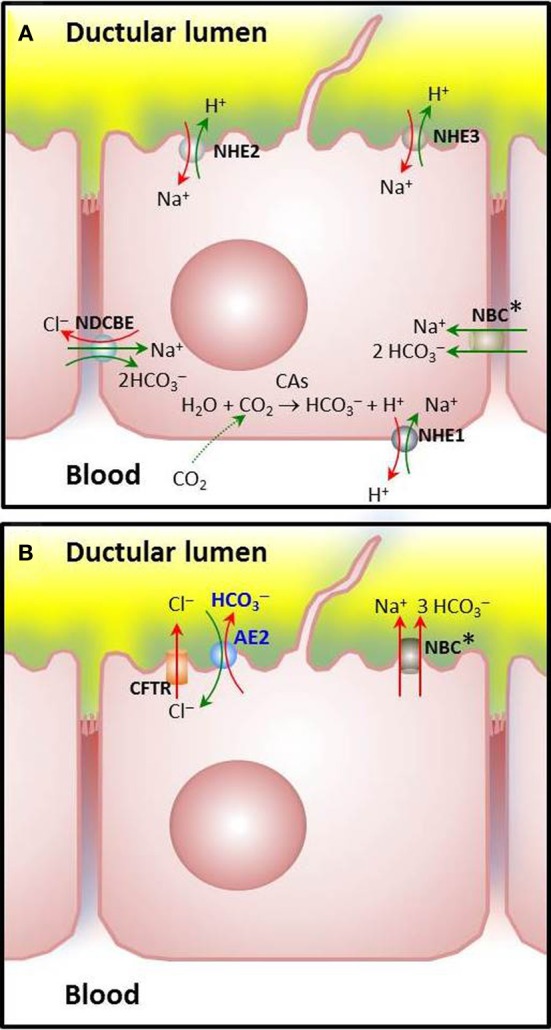
**Major ion carriers involved in pH_i_ regulation in cholangiocytes**. **(A)** Acid extruders or HCO^−^_3_ loaders: Cells are loaded with HCO^−^_3_ via CO_2_ hydration catalyzed by carbonic anhydrases [CO_2(g)_+H_2_O_(l)_ ↔ HCO_3−(aq)_ + H^+^_(aq_)] and subsequent H^+^ extrusion through Na^+^/H^+^ exchange, mainly mediated by the basolateral amiloride-sensitive NHE1, that is recognized as a potent acid extruder. Amiloride-insensitive NHE2 and amiloride-sensitive NHE3 may also participate, though these apical exchangers seems to play a major role for NaCl and fluid absorption from the bile duct lumen (Strazzabosco, 1997; Spirlì et al., 1998; Mennone et al., 2001). Additionally, Na^+^: HCO^−^_3_ cotransporters (NBC) with a stoichiometry of 1:2 or a Na^+^-dependent Cl^−^/HCO^−^_3_ exchanger (NDCBE) may load HCO^−^_3_ (in rodent or human cholangiocytes, respectively). **(B)** Acid loaders: The Na^+^-independent Cl^−^/HCO^−^_3_ exchanger AE2 is the major acid loader in cholangiocytes. Physiologically, it extrudes HCO^−^_3_ in exchange with Cl^−^ once a high outside to inside gradient has been established following stimulation of a variety of apical Cl^−^ channels (the cAMP-activated CFTR and the Ca^2+^-dependent TMEM16A—illustrated in Figure 2—among other channels pending a complete characterization). Characteristically, mouse cholangiocytes possess an additional capability for acid loading through Na^+^: HCO^−^_3_ cotransport (putatively with a stoichiometry of 1:3) (Uriarte et al., 2010). The biliary epithelial cells have other ion carriers like those for Cl^−^, Na^+^, and K^+^ (not shown) which may contribute, at least indirectly, to pH_i_ regulation and/or HCO^−^_3_ secretion. Asterisks are used to indicate that locations for NBC(s) remain to be definitely determined.

Cholangiocytes are crucial for modifying the primary bile generated at the canaliculi, as they are capable of secretory and absorptive functions that results in bile fluidization and alkalinization (Tabibian et al., 2013). Under physiological conditions, a major function of cholangiocytes is the biliary secretion of HCO^−^_3_ (Banales et al., 2006b). As illustrated in Figure 1, cholangiocytes may accumulate HCO^−^_3_ through direct HCO^−^_3_ loading from the basolateral membrane and/or by *ex-novo* HCO^−^_3_ generation upon hydration of CO_2_ and subsequent H^+^ extrusion (Tabibian et al., 2013). CO_2_ hydration is catalyzed by carbonic anhydrases (CAs) (Alterio et al., 2012), several isoforms of which are expressed in the biliary tract. The cytosolic carbonic anhydrase type II (CA-II) is highly expressed in cholangiocytes and seems to be the major isoform participating in the *ex-novo* generation of HCO^−^_3_ (Tabibian et al., 2013). Membrane-bound CA-IV and CA-IX were localized in the biliary tract and could also participate in the process (Kivela et al., 2005).

**Figure 2:**
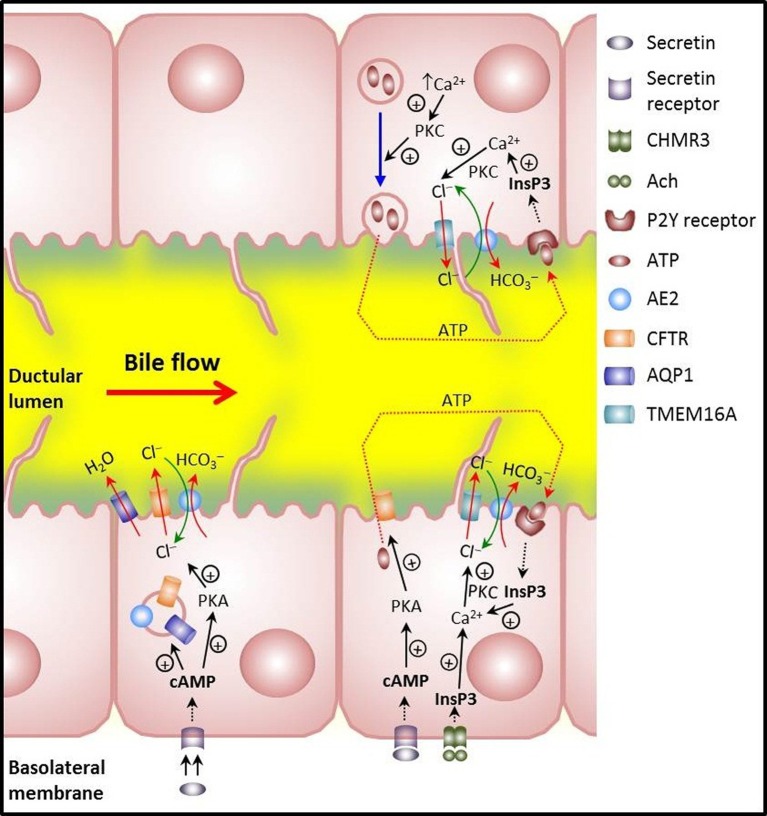
**Main mechanisms involved in biliary HCO^−^_3_ secretion in cholangiocytes**. *Lower left*: illustrates that the hormone secretin induces trafficking of vesicles with the chloride channel CFTR, the anion exchanger AE2/SLC4A2, and the water channel AQP1. Vesicle exocytosis at the apical membrane allows for bicarbonate-rich hydrocholeresis. *Lower right*: cholinergic stimulation of basolateral M3 muscarinic receptors increases InsP3 and leads to Ca^2+^ release. Activation of the apical Ca^2+^-dependent Cl^−^ channel TMEM16A results in efflux of Cl^−^ which is then exchanged with HCO^−^_3_ via AE2. Moreover, CFTR activation that follows secretin stimulation may induce apical release of ATP with further stimulation of apical P2Y receptors, increases in InsP3 and Ca^2+^, activation of apical Ca^2+^-dependent Cl^−^ channel TMEM16A, Cl^−^ efflux and final AE2-mediated Cl^−^/HCO^−^_3_ exchanger for apical HCO^−^_3_ secretion. *Upper right*: release of ATP that follows PKC-dependent exocytosis of ATP-enriched intracellular vesicles upon increases in cell volume. Further stimulation of apical P2Y receptors may end up with apical HCO^−^_3_ secretion as described for the CFTR-dependent release of ATP.

One of the mechanisms by which HCO^−^_3_ is secreted from cholangiocytes involves activation of Cl^−^ channels and efflux of Cl^−^ followed by its exchange with HCO^−^_3_ (Figure 2) (Alvaro et al., 1993; Banales et al., 2006b; Tabibian et al., 2013). But in order to maintain the overall HCO^−^_3_-secretory function, cholangiocytes are provided with specific ion membrane carriers like acid loaders and acid extruders (Figure 1) which allow them to maintain ion gradients and pH_i_ (Strazzabosco et al., 1991; Banales et al., 2006b; Tabibian et al., 2013).

### Acid extruders in cholangiocytes

#### Na^+^/H^+^ exchangers (NHEs)

The extrusion of H^+^ occurs through Na^+^/H^+^ exchangers (Donowitz et al., 2013). NHE1-3/SLC9A1-3—the three major isoforms reported in rat cholangiocytes—differ in their functional properties, sensitivity to inhibitors, regulatory mechanisms, and/or membrane polarity (see Figure 1A and references therein). The basolateral NHE1 protects cholangiocytes from intracellular H^+^ accumulation due to cell metabolism, *ex-novo* generation of H^+^ after CO_2_ hydration, and additional ion transport. Thus, NHE1 plays a crucial role for pH_i_ homeostasis (in combination with diverse HCO^−^_3_ transporting systems like Na^+^- HCO^−^_3_ cotransporters and Na^+^-dependent and Na^+^-independent Cl^−^/HCO^−^_3_ exchangers), while the apical acid extruders NHE2 and NHE3 seems to play an important role for NaCl and fluid absorption from the bile duct lumen (see also references in Figure 1A).

#### Na^+^-bicarbonate cotransporters (NBCs)

The mechanisms leading to increased intracellular concentration of HCO^−^_3_ in cholangiocytes (through HCO^−^_3_ influx or *ex-novo* generation) all function as acid extruders. In rodent cholangiocytes HCO^−^_3_ influx is known to be mediated by Na^+^- HCO^−^_3_ cotransport (Strazzabosco et al., 1991). This HCO^−^_3_-loading function has been demonstrated for an isoform of the electrogenic Na^+^- HCO^−^_3_ cotransporter NBC1 (also referred to as SLC4A4 and NBCe1) expressed in the basolateral membrane of pancreatic ducts. This variant pNBC1 was found to mediate Na^+^ and HCO^−^_3_ influx by coupling the transport of 2 HCO^−^_3_ to the downhill flux of Na^+^, i.e., by operating with a Na^+^: HCO^−^_3_ stoichiometry of 1:2 (Gross and Kurtz, 2002). But the stoichiometry of NBC1 and the direction of the transmembrane Na^+^- HCO^−^_3_ cotransport may vary from one cell type to another (Gross and Kurtz, 2002). Also, it may change in the same cell depending on the intracellular levels of cAMP and PKA-dependent phosphorylation of the COOH terminus, that favor the 1:2 over the 1:3 stoichiometry (Gross and Kurtz, 2002; Pushkin and Kurtz, 2006) and on the intracellular concentration of calcium [Ca^2+^]_i_, the increase of which operates in the opposite direction (see below for NBC1 as a potential acid loader).

#### Na^+^-driven CL^−^/HCO^−^_3_ exchangers (NDCBEs)

In human cholangiocytes, the Na^+^- HCO^−^_3_ cotransport appears to be inactive at physiological pH, and HCO^−^_3_ influx is carried out through electroneutral Na^+^-dependent Cl^−^/HCO^−^_3_ anion exchange (Strazzabosco et al., 1997), which functions via the uptake of one Na^+^ and the equivalent of 2 HCO^−^_3_, together with the efflux of one Cl^−^ (Romero et al., 2004). SLC4A8 is the only Na^+^-dependent Cl^−^/HCO^−^_3_ exchanger cloned in humans so far. Although early experiments of Northern blot failed to detect the SLC4A8 mRNA in whole liver (Grichtchenko et al., 2001), *SLC4A8* expression cannot be discarded in cholangiocytes which represent only 5% of the liver cell population.

### Acid loaders in cholangiocytes

The major acid-loading mechanism in cholangiocytes involves an apical electroneutral and Na^+^-independent Cl^−^/HCO^−^_3_ exchange (Strazzabosco et al., 1991; Spirlì et al., 1998) (Figure 1B). Since the direction of such an exchange is determined by the transmembrane gradient and the outside to inside gradient of Cl^−^ is higher, the exchange normally functions secreting HCO^−^_3_ (Banales et al., 2006a,b). Several members of SLC4 and SLC26 families (SLC4A1, SLC4A2, SLC4A3 and SLC26A3, SLC26A4, and SLC26A6) could work in this regard (Pushkin and Kurtz, 2006; Dorwart et al., 2008). However, only SLC4A2 (also AE2) was encountered in cholangiocytes (see Medina, 2011). Moreover, *AE2*-knockdown experiments in human and rat cholangiocytes indicated that AE2 is the main effector of their Cl^−^/HCO^−^_3_ exchange activity (Banales et al., 2006a, 2012; Arenas et al., 2008). Also, *Ae2*^−/−^_*a,b*_ mouse cholangiocytes, but not wildtype-control cells, lacked Cl^−^/HCO^−^_3_ exchange activity (Uriarte et al., 2010). Characteristically, mouse cholangiocytes exhibited electrogenic NBC activity and NBC1 expression that was increased in *Ae2*^−/−^_*a,b*_ cholangiocytes (Uriarte et al., 2010). Ae2-deficient cells also showed elevated [Ca^2+^]_*i*_, known to favor the 1:3 stoichiometry of NBC1 that promotes an extruding Na^+^: HCO^−^_3_ cotransport (Muller-Berger et al., 2001). These data suggest that overexpressed NBC1 might attempt to replace AE2 for HCO^−^_3_ extrusion and intracellular acidification in the knockout cholangiocytes.

Another Na^+^- HCO^−^_3_ cotransporter, referred to as NBC4 (also SLC4A5/NBCe2) was reported to be expressed in cholangiocytes. Similarly to NBC1, NBC4 is electrogenic and may work with 1:3 and 1:2 stoichiometries. Among the six NBC4 splice variants (NBC4a–f) identified in humans, only the NBC4c variant was found to be expressed in rat liver, being basolateral in hepatocytes but apical in cholangiocytes (Abuladze et al., 2004; Pushkin and Kurtz, 2006). In human cholangiocytes, however, the Na^+^-HCO^−^_3_ cotransport mechanism is seemingly inactive at physiological pH_i_ (Strazzabosco et al., 1997; Uriarte et al., 2010) and AE2 represents their major acid-loading mechanism.

### Other ion transporters in cholangiocytes

Cholangiocytes are equipped with additional ion transporters like those for Cl^−^, Na^+^, and K^+^, that may contribute at least indirectly, to pH_i_ regulation. At the apical pole, for instance, cholangiocytes express not only the cAMP-responsive Cl^−^ channel CFTR that is activated by secretin (SCT), but also the mechanosensitive and Ca^2+^-activated Cl^−^channel TMEM16A and other Ca^2+^- and/or PKC-dependent Cl^−^ channels (Dutta et al., 2011, 2013). These Ca^2+^-dependent channels may contribute to Cl^−^ secretion in response to diverse Ca^2+^-dependent stimuli such as ATP stimulation and cell volume increase. Cl^−^efflux is important for the outside to inside Cl^−^ gradient that facilitates the electroneutral Cl^−^/HCO^−^_3_ exchange via AE2. Though this exchange partially restores intracellular Cl^−^ levels, the basolateral Na^+^-K^+^-2 Cl^−^ cotransporter NKCC1 (also referred to as SLC12A2) can allow for further Cl^−^ influx accompanied by Na^+^ and K^+^ (Singh et al., 2001). A basolateral sodium pump Na^+^/K^+^ ATPase may achieve the efflux of 3 Na^+^ by the influx of 2 K^+^ (Rakowski et al., 1989; Scoazec et al., 1997), while cAMP and/or Ca^2+^-sensitive basolateral K^+^ channels can mediate K^+^ extrusion. The small conductance K^+^ channel SK2/KCNN2 has been detected in cholangiocytes both apical and basolateral, although functional studies revealed greater basolateral Ca^2+^-stimulated K^+^ conductance (Feranchak et al., 2004). The osmotic gradient generated in the bile duct lumen drives secretion of water via aquaporin 1 (AQP1) (Marinelli et al., 1999). Interestingly, AQP1 colocalizes with CFTR and AE2 in subapical vesicles that are exocytosed into the apical membrane upon cAMP-related stimulation (Tietz et al., 2003, 2006), which supports a coordinated contribution of these transporters for the generation of ductal bile flow. Finally, basolateral AQP4 makes possible transcellular water flux for bile fluidization by mediating the import of water from the peribiliary vascular plexus surrounding the bile duct (Marinelli et al., 2000).

## The role of AE2 for biliary HCO^−^_3_ secretion

Bicarbonate transporters of the SLC26 family that display cAMP- and Ca^2+^-dependent electroneutral Cl^−^/HCO^−^_3_ exchange (Romero et al., 2004; Garnett et al., 2011; Rode et al., 2011) have been reported to drive HCO^−^_3_ secretion in several epithelia. This is the case, for instance, in pancreatic and salivary glands, where those exchangers mediate apical HCO^−^_3_ secretion while AE2 is seemingly intended to regulate pH_i_ changes from the basolateral site (Vázquez et al., 1995; Lee et al., 2012). But in the liver there is no evidence for the expression of any of those SLC26 transporters and AE2 appears to be the only operative Cl^−^/HCO^−^_3_ exchanger in the hepatobiliary cells. Compatible with its ability to secrete HCO^−^_3_ into bile, AE2 was localized with a monoclonal antibody at the apical domain of both cholangiocytes and hepatocytes (Martínez-Ansó et al., 1994; Medina et al., 1997). On the other hand, and in agreement with previous reports (Alper, 2009), the same antibody localized AE2 at the basolateral domain in choroid plexus (Martínez-Ansó et al., 1994), salivary glands (Vázquez et al., 1995), and kidney (Castillo et al., 2000). The characteristic apical targeting of AE2 in liver cells is further supported by data obtained in different rat models (Tietz et al., 2003; Aranda et al., 2004; Banales et al., 2008; Úriz et al., 2011).

In addition to the aforementioned *AE2*-knockdown experiments in human and rat cholangiocytes and assessments in Ae2-knockout mouse cholangiocytes indicating that AE2 is by far the major Cl^−^/HCO^−^_3_ exchanger in these biliary cells (see references above), *in situ* hybridization in human liver further confirmed that the *AE2* gene is extensively expressed in cholangiocytes (García et al., 1998). Thus, AE2 is currently regarded as the Cl^−^/HCO^−^_3_ exchanger cholangiocytes have not only for pH_i_ regulation but also as a key HCO^−^_3_ extruder (in close interaction with CFTR and other Cl^−^ channels), for HCO^−^_3_ secretion to bile.

Biliary HCO^−^_3_ secretion is tightly regulated by local factors such as bile salts and purinergic agonists, particularly the potent secretagogue adenosine triphosphate (ATP) and by visceral neurohormonal factors including cholinergic and adrenergic agents, vasoactive intestinal peptide (VIP), glucagon, glucagon-like peptide-1, somatostatin and, above all, SCT (Alvaro et al., 2007; Beuers et al., 2010). In the case of SCT (see Figure 2), the interaction of the hormone with its receptor SCTR at the basolateral membrane of cholangiocytes results in functional stimulation of the SCT/SCTR/CFTR/Cl^−^/HCO^−^_3_—AE2 system (Úriz et al., 2011; Afroze et al., 2013) by increasing cAMP levels and protein kinase A activation (Alvaro et al., 1997b). Subsequent mobilization of AE2/CFTR/AQP1-containing intracellular vesicles toward the apical membrane is followed by vesicle endocytosis (Tietz et al., 2003) (Figure 2). Accompanying phosphorylation and activation of CFTR leads to apical efflux of Cl^−^ that is ultimately exchanged with HCO^−^_3_ through AE2 (Alvaro et al., 1997b; Banales et al., 2006a,b). Moreover, increased cAMP levels can stimulate the AE activity in cholangicytes (Spirlì et al., 1998; Zsembery et al., 1998).

The choleretic effect of an increase in the levels of cAMP may be enhanced through a CFTR-associated release of ATP from cholangiocytes into bile (Minagawa et al., 2007) and autocrine/paracrine stimulation of apical purinergic P2Y receptors. The resultant increase in intracellular Ca^2+^ can activate the apical Ca^2+^-dependent Cl^−^ channel TMEM16A (Dutta et al., 2011), promoting additional efflux of Cl^−^ that will be exchanged with HCO^−^_3_ through AE2. Ursodeoxycholic acid (UDCA) is also able to stimulate CFTR-associated biliary ATP release leading to purinergic stimulation, [Ca^2+^]_i_ increase and PKC activation, Cl^−^ efflux and AE2-mediated exchange with HCO^−^_3_ (Fiorotto et al., 2007). Moreover, ATP may be released upon exocytosis of ATP-enriched intracellular vesicles in response to increases in cell volume in a PKC-dependent manner (Sathe et al., 2011). As mentioned above for the CFTR-associated ATP release, this type of purinergic stimulation—seemingly mediated by the vesicular nucleotide transporter SLC17A9—is expected to result in further Cl^−^ efflux through TMEM16A. Whether the SLC17A9-dependent exocytosis of ATP-enriched intracellular vesicles and the CFTR-associated ATP release are closely related remains yet to be determined.

Acetylcholine and cholinergic stimulation may further assist biliary HCO^−^_3_ secretion through Ca^2+^-dependent Cl^−^ efflux from cholangiocytes, thus potentiating the effect of SCT on both intracellular cAMP levels and Cl^−^/HCO^−^_3_ exchange in a calcineurin-dependent manner (Alvaro et al., 1997a; Minagawa et al., 2007).

All these data indicate the existence of a close mechanistic interplay between the cAMP/PKA and [Ca^2+^]_i_/PKC pathways when potent secretagogues exert their choleretic effect. A common mechanistic endpoint appears to be an increase in biliary Cl^−^ efflux, and the fact that such an increase leads to augmented biliary HCO^−^_3_ secretion indicates that AE2-mediated Cl^−^/HCO^−^_3_ exchange is critical for the enhanced choleresis to occur.

AE2 interactions with cytosolic and/or membrane bound CAs, similar to those described for cells other than biliary cells (Sterling et al., 2001, 2002; Morgan et al., 2007), may also contribute to AE2-mediated biliary HCO^−^_3_ secretion.

## How AE2 deficiency may lead to PBC?

PBC is a cholestatic liver disease of unknown etiopathogenesis which affects mainly middle-aged women and concurs with characteristic autoimmune phenomena (Hohenester et al., 2009; Poupon, 2010). In the early 90s we hypothesized that PBC pathogenesis could be related to alterations in the mechanisms of HCO^−^_3_ transport because: (i) most PBC patients improve the clinical course of the disease by continued treatment with UDCA (Corpechot et al., 2005); and (ii) the hydrophilic bile acid UDCA is known to produce a HCO^−^_3_-rich hydrocholeresis [see Medina (2011), for a review]. Interestingly, we could find reduced expression of AE2 in liver biopsies and peripheral blood mononuclear cells from patients with PBC (Prieto et al., 1993; Medina et al., 1997). Also, human cholangiocytes isolated from PBC patients showed a decreased response of the AE activity to cAMP stimulation (Melero et al., 2002), and more recent findings pointed to microRNA-506 being upregulated in cholangiocytes from PBC patients (Banales et al., 2012). This microRNA may bind to the 3'UTR region of AE2 mRNA and prevent protein translation leading to diminished AE2 activity (Banales et al., 2012). Additionally, many immunologic and hepatobiliary alterations characteristic of PBC are eventually reproduced in *Ae2*^−/−^_*a,b*_ mice which indeed develop both immunologic and hepatobiliary PBC-like alterations (Salas et al., 2008). Interestingly, cholangiocytes isolated from these *Ae2*^−/−^_*a,b*_ mice show no increase in resting pH_i_ despite the AE2 deficiency, most probably because of their ability to upregulate the aforementioned NBC activity with acidifying potential (Uriarte et al., 2010). This acidifying cotransport activity is absent in human cholangiocytes (Arenas et al., 2008). Complete deficiency of AE2 would therefore be expected to result in intracellular alkalinization, but most PBC patients show diminished (rather than absent) AE2 expression (Medina et al., 1997), and residual AE2 activity may allow them to maintain normal resting pH_i_ (Melero et al., 2002). In this regard, it can be assumed that upon situations of stimulated hydroionic transport, pH_i_ homeostasis might undergo parallel abnormalities in human cholangiocytes from PBC patients and mouse cholangiocytes from *Ae2*^−/−^_*a,b*_ mice.

Recently, Beuers et al. postulated a new and attractive hypothesis that may explain how AE2 deficiency could contribute to the pathogenesis of PBC and other human cholangiopathies. This hypothesis proposes that biliary epithelial cells develop a HCO^−^_3_ umbrella at their luminal membrane to protect themselves against bile-salt induced injury (Beuers et al., 2010). By maintaining an alkaline environment around the luminal membrane of cholangiocytes, the protonation of apolar hydrophobic bile salt monomers could be prevented, rendering those monomers unable to permeate membranes in an uncontrolled fashion and avoiding toxic effects on cholangiocytes. In a series of elegant experiments, the authors then demonstrated that an intact glycocalix at the apical membrane of cholangiocytes and adequate AE2 expression are crucial to maintain the biliary HCO^−^_3_ umbrella (Hohenester et al., 2012). Therefore, it appears that cholangiocytes use AE2 as a highly beneficial *two-edged sword* which allows them to fulfill: (i) the direct control of the pH_i_ preventing, for instance, that any ATP-stimulated increase in NHE activity (Zsembery et al., 1998; Melero et al., 2002) leads to harmful intracellular alkalinization; and (ii) the immediate generation of the apical alkaline umbrella, preventing highly concentrated hydrophobic bile salt monomers to enter the cell.

In the case of PBC, dysfunctional lymphocytes play a crucial role in the pathogenesis of the disease. Of note, our *Ae2*^−/−^_*a,b*_ mouse model (Salas et al., 2008) supports the view that these immune cells require AE2 for controlling their protective surveillance in a way that tolerance is preserved and autoimmunity does not come out. Peripheral blood mononuclear cells from PBC patients exhibit a decrease in AE2 (Prieto et al., 1993), and therefore the risk for a break of tolerance may be increased. In summary, diminished AE2 activity in cholangiocytes from PBC patients may lead to cell injury that makes them more provoking to the immune system. Additionally, diminished AE2 activity in PBC lymphocytes may contribute to these cells being more aggressive toward the provoking cholangiocytes, resulting in profound damage of the biliary tree.

### Conflict of interest statement

The authors declare that the research was conducted in the absence of any commercial or financial relationships that could be construed as a potential conflict of interest.
